# The history of Down syndrome–associated Alzheimer's disease; past, present, and future

**DOI:** 10.1002/alz.70158

**Published:** 2025-06-05

**Authors:** Lucia Maure‐Blesa, María Carmona‐Iragui, Ira Lott, Elizabeth Head, Thomas Wisniewski, Michael S. Rafii, Joaquín Espinosa, Jesús Flórez, William C. Mobley, Anthony Holland, André Strydom, Shahid Zaman, Juan Fortea

**Affiliations:** ^1^ Sant Pau Memory Unit Department of Neurology Hospital de la Santa Creu i Sant Pau Biomedical Research Institute Sant Pau Universitat Autònoma de Barcelona, Departamento de Medicina Barcelona Spain; ^2^ Center of Biomedical Investigation Network for Neurodegenerative Diseases (CIBERNED), Alzheimer's disease and other neurodegenerative dementias Madrid Spain; ^3^ Barcelona Down Medical Center Fundació Catalana de Síndrome de Down Barcelona Spain; ^4^ Institute for Memory Impairments and Neurological Disorders (MIND) University of California, Irvine Irvine California USA; ^5^ Department of Pediatrics University of California, Irvine School of Medicine Orange California USA; ^6^ Department of Pathology and Laboratory Medicine University of California, Irvine Irvine California USA; ^7^ Center for Cognitive Neurology Department of Neurology New York University Grossman School of Medicine New York New York USA; ^8^ Department of Pathology and Department of Psychiatry New York University Grossman School of Medicine New York New York USA; ^9^ Alzheimer's Therapeutic Research Institute Keck School of Medicine University of Southern California San Diego California USA; ^10^ Linda Crnic Institute for Down Syndrome University of Colorado Anschutz Medical Campus Aurora Colorado USA; ^11^ Department of Pharmacology University of Colorado Anschutz Medical Campus Aurora Colorado USA; ^12^ Department of Physiology and Pharmacology University of Cantabria Santander Spain; ^13^ Department of Neurosciences University of California San Diego La Jolla California USA; ^14^ Cambridge Intellectual and Developmental Disabilities Research Group Department of Psychiatry University of Cambridge Cambridge UK; ^15^ Department of Forensic and Neurodevelopmental Sciences Institute of Psychiatry Psychology & Neuroscience King's College London London UK

**Keywords:** Alzheimer's disease, amyloid, clinical trials, Down syndrome, Down syndrome–associated Alzheimer's disease, historical perspective

## Abstract

The landscape of Down syndrome–associated Alzheimer's disease (DSAD) research reflects decades of scientific endeavor and collaborative effort, charting a remarkable journey from initial observations to the elucidation of complex genetic and molecular mechanisms. This perspective article chronicles key milestones and breakthroughs, paying homage to the pioneering scientists and advancements that have shaped the field. A thorough review of historical and contemporary literature offers a comprehensive narrative, highlighting the evolution of knowledge surrounding DSAD, from early recognition to the characterization of clinical presentation and natural history. The unique challenges and ethical considerations associated with DSAD populations are also examined, underscoring the importance of tailoring research and clinical approaches. By reflecting on the field's trajectory, this work celebrates past achievements while emphasizing the critical need for sustained research efforts. As part of a special issue, this article provides a foundation for appreciating the challenges and opportunities that lie ahead in advancing DSAD understanding and care.

**Highlights:**

- This article provides a comprehensive overview of Down syndrome–associated Alzheimer's disease (DSAD) history, from early descriptions to its recognition as a genetic form of AD.
- It reflects on historical challenges faced by individuals with intellectual disabilities in achieving inclusion in scientific research.
- This historical perspective highlights the critical contributions of individuals with DS in advancing understanding of AD natural history.
- It explores pivotal milestones and efforts that have driven progress in DSAD research.
- Finally, it provides context to understand challenges and opportunities in DSAD research and its future directions.

## 1 INTRODUCTION

More than 150 years after Down syndrome (DS) was first described, advancements in health care have significantly improved life expectancy, now reaching 60 years in many countries of the Global North. This progress has shifted Alzheimer's disease (AD) in people with DS to the forefront as it represents a major medical challenge and life‐limiting factor in this population. Over the past several decades and more recently, research for people with DS has been pioneering innovation, but at other times has been strikingly underserved and underrepresented. Currently, with increased attention, investment, breakthrough research, and promising clinical trials, DS‐associated AD (DSAD) research is poised to hold a pivotal position in AD research at large. In this special issue, we reflect on the historical and scientific milestones of DS and DSAD, highlighting contributions that advance our broader understanding of AD.

As we examine this history, it is essential to acknowledge the power of language in shaping both scientific discourse and societal attitudes. Historically, people with DS and other intellectual disabilities (IDs) have faced systemic discrimination, and language has often been a tool of exclusion and harm. While this review references terminology used in historical contexts for accuracy, we firmly recognize that some of these terms are now unequivocally inappropriate. The scientific community bears a responsibility not only to advance knowledge but also to uphold the dignity and rights of the populations we study. As language evolves, so must our commitment to using respectful, affirming, and person‐centered terminology—both to reflect contemporary standards and to foster a more inclusive and just scientific field.

## 2 HISTORICAL BACKGROUND OF DS

### 2.1 An integral part of humanity since its origins

Recent discoveries suggest that people with DS have been an integral part of human history since prehistoric times. For instance, research by Conde‐Valverde et al.^1^ identified what is likely the earliest known individual with DS: a Neanderthal child who lived ≈ 143,000 to 271,000 years ago. Remarkably, this child survived to the age of six, an impressive milestone that reflects care and support from the community.

The oldest cases of individuals with DS in our human species have been documented in different populations including Neolithic Ireland (≈ 3500 BCE), Bronze Age Bulgaria (≈ 2700 BCE) and Greece (≈ 1300 BCE), Iron Age Spain (≈ 600 BCE), or Post‐Medieval Finland (≈ 1720 CE).^2^ In all cases, evidence shows that ancient societies likely took care and acknowledged these individuals as members of their communities, as all reported burials of these infants with DS were either special, or performed with care according to standard practices.^2^

The concept of inclusion and care for individuals with disabilities, including DS, as an inherent aspect of human nature is also reinforced by anthropological evidence. Artistic depictions of individuals with DS appear throughout history.^3^, ^4^ The earliest representation may be a figurine from Greece dating back to ≈ 5000 BCE.^4^ Since then, subsequent cultures have represented individuals with probable DS, including the Olmecs living near the Gulf of Mexico (≈ 1500 BC) in figurines and murals,^4^ ancient Egyptians (≈ 100 AD) and notable European artworks such as the Virgin and Child with Saints Jerome and Louis of Toulouse painting (≈ 1455 AD), the Ecce Homo scene painting (≈ 1505 AD), and the paintings by the Flemish painter Jacob Jordaens (1593–1678).^4^

However, throughout history, the integration of individuals with IDs in social life; education; health care; and, ultimately, scientific research has been a complex and evolving process marked by ongoing struggles and significant shifts, as we will touch upon in this article.

### 2.2 The identification of DS as a specific form of ID

Although all historical evidence demonstrates that the people with DS existed prior to the nineteenth century, it was not until then that the different etiologies of ID were described, and DS recognized as a distinct form of ID.

The French psychiatrist Jean‐Etienne Dominique Esquirol published in 1838 one of the first handbooks of psychiatry *Des maladies mentales considérées sous les rapports médical, hygiénique et médico‐légal [Mental Disease: Medical, Health/Hygiene and Medical‐Legal Considerations*],^5^ in which a large section was devoted to “idiocy,” recognizing for the first time individuals with ID as a different entity than psychiatric conditions.

A few years later, Esquirol's former student and French physician Edouard Séguin published a series of pivotal works (*Traitement moral, hygiene et education des Idiots* [1846]^6^) regarding education and care of individuals with IDs. In his work, partially inspired by Dr. Itard's prior seminal publication^7^ on social education, Dr. Séguin underscored the importance of socialization and educational methodologies for individuals with developmental challenges, which had a significant impact on how society approached all forms of disabilities. He also took up the work started by Dr. Esquirol and classified the distinct forms of “idiocy” based on physical appearance, cognitive and social behavior, and the ability to learn, identifying a group with “(…)the nostrils flattened and small, the eyes dull and immobile, the hair soft and often sparse, the forehead low, (…) generally imitative, possesses memory, cheerfulness, timidity, grace, and often mischief.”^6^

In 1866, Dr. John Langdon Down, a clinician that was at the time working in an “asylum for idiots” in England, reported a detailed description of a group that exhibited a characteristic syndrome. Due to the consistent resemblance of certain facial features with the appearance of the Kalmyk people, a Mongolic ethnic group,^8^, ^9^ he coined the term “Mongolian idiocy” or “Kalmuc.” Only a decade later, Fraser and Mitchell published a detailed description of 62 individuals with this condition, highlighting, among other features, the early onset of “senility” in this population: “I think it nearly certain that they are short‐lived (…), in not a few instances, however, death was attributed to nothing more definite than general decay—a sort of precipitated senility.”^10^

Several hypotheses were considered regarding the origin of the syndrome, including parental tuberculosis (Down et al.^8^), geographic location, or a common ethnic background. However, it wasn't until the development of cytogenetic techniques and the discovery of the human genome^11^ that the genetic cause of DS was established.

The identification of chromosomal trisomy as the cause of DS was first reported in 1959 by French geneticist Jérôme Lejeune and his colleagues Marthe Gautier and Raymond Turpin.^12^ Subsequently, these findings were independently confirmed by British researchers Patricia Jacobs and John Strong^13^ and Swedish researchers J.A. Böök, M. Fraccaro, and J. Lindsten.^14^ Trisomy was identified for chromosome 21 and officially named as such during the 1960 Denver Conference.

In line with the prevailing views of the medical community and researchers, the term “mongolism,” considered outdated and offensive, changed to “Down syndrome” during the 1960s.^15^

## 3 HOW DS CONTRIBUTED TO THE CHARACTERIZATION OF AD

### 3.1 Pathological and histochemical similarities

In 1929, two decades after Alois Alzheimer´s seminal publication and his and Oskar Fischer's clinicopathological description of the neuritic plaques in the brains of individuals with dementia,^16^, ^17^, ^18^ Dr. Struwe reported for the first time the presence of these “senile plaques” in the brain of a 37‐year‐old “mongoloid” patient.^19^ This finding gained even more significance when accompanied by the description of not only neuropathological but also clinical similarities: Fraser and Mitchell had already remarked in 1876 on their “early onset of senility,”^10^ and Jervis published in 1948 a detailed article highlighting symptoms of cognitive decline and dementia in individuals with DS at very early ages “[with the] exception of the age of onset, the clinical and pathological manifestations are those of senile dementia (…) in the few mongoloid idiots who reach the fourth or fifth decade of life, remarkable personality changes may occur, resulting from intellectual and emotional deterioration. In these patients, the underlying brain lesions are those of pathological senility.”^20^ Although at this point the cause of the syndrome was still unknown, Jervis considered studying its relationship with dementia: “since mongoloid patients show a marked tendency to develop this type of reaction, it is suggested that the study of it offers some information which may contribute to a better understanding of the causes of senile dementia.”^20^

**FIGURE 1:**
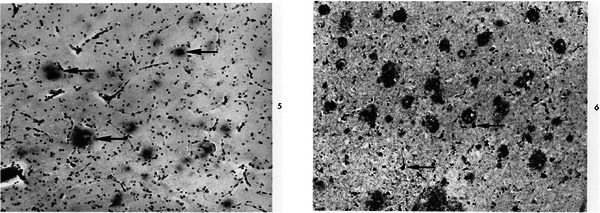
Images taken from the paper by Burger and Vogel^22^ showing AD pathological changes in brain samples from individuals with DS: "5: Scattered senile plaques (arrows) in the frontal lobe of case 2 (17 years) consist of loose aggregates of argyrophilic neurites (King silver stain, x 120). 6: Many dense senile plaques, as well as neurons showing neurofibrillary change (arrows) are present in the frontal lobe of case 8 (53 years) (King silver stain, x 120)", with permission from Elsevier. AD, Alzheimer's disease; DS, Down syndrome.

The comparison of the pathology in individuals with DS and dementia and individuals with AD in the general population showed the same neuropathological hallmarks of the disease. These lesions developed at much younger ages in all individuals with DS and in a sequential manner; Malamud^21^ and Burger and Vogel^22^ (Figure 1) proved in their respective research that all individuals with DS above the fourth decade had the full‐blown AD pathology in their brains irrespective of symptoms.^21^, ^22^, ^23^, ^24^, ^25^

These neuropathology studies suggested a common underlying mechanism, but whether people with DS had in general a “more rapid aging process” or whether their specific genetic background was directly causing AD pathology was a matter of debate.^21^, ^22^ The possibility that a potential gene dose effect of a gene coded in chromosome 21 could explain the matter was suggested:^25^ “We have studied the autopsy findings of a series of patients with Down's syndrome, with a view toward evaluating this natural predilection as a source of information about the pathogenesis and etiology of Alzheimer's disease and dementia (…) Although the findings do not offer an understanding of the pathogenesis of these disorders, they suggest that the etiologic factors be sought in the genotypic and/or phenotypic setting of Down's syndrome.”^22^

**FIGURE 2:**
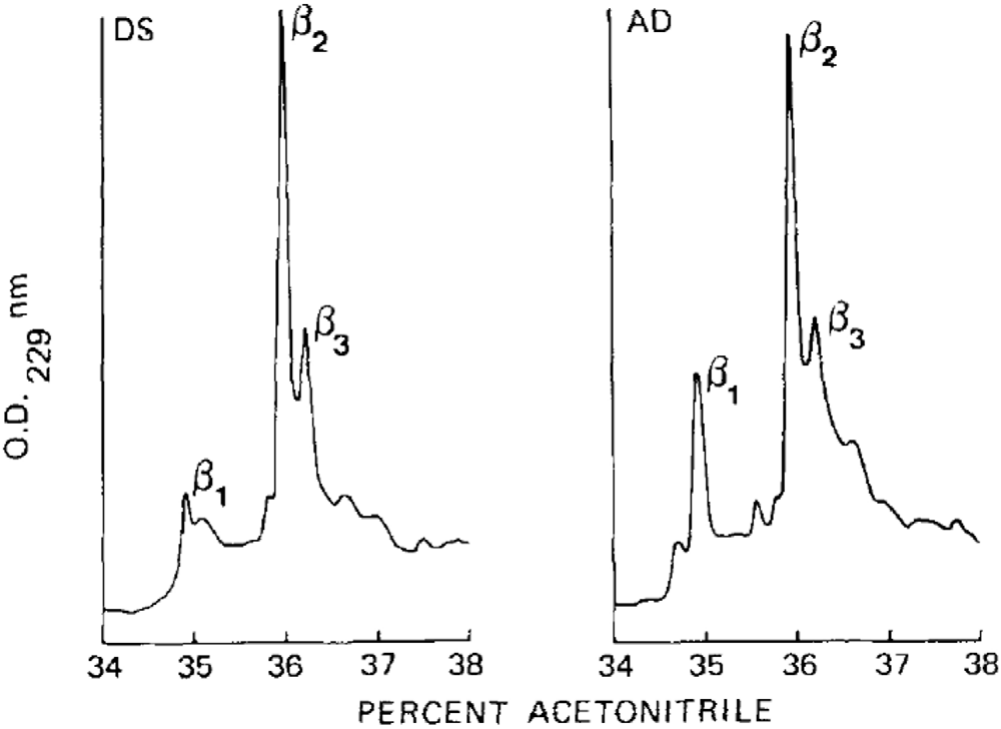
Image taken from “Glenner GG, Wong CW. Alzheimer's disease and Down's syndrome: sharing of a unique cerebrovascular amyloid fibril protein. Biochem Biophys Res Commun. 1984”^27^ with permission from Elsevier “High Performance Liquid Chromatography (HPLC) of the cerebrovascular amyloid fibril β protein from an Alzheimer's disease patient (AD) previously isolated on Sephadex G‐100 as compared to the β protein of an adult Down's syndrome individual (DS) demonstrating three major protein peaks: β1, β2 and β3. The β1 and β2 proteins have identical amino‐terminal amino acid sequences, while the characteristics of β3 are presently unknown”.

In 1984 and 1985, with the development of biochemical techniques, Glenner and Wong^26^ purified the primary amyloid beta (Aβ) protein of neuritic plaques and also cerebral vessels in the brains of euploid patients with AD. They found no homology between this protein and any protein sequenced thus far, establishing it as a biologic marker for the cerebrovascular amyloid fibril component of AD. Soon after, Aβ protein was also isolated from the brain of one individual with DS and dementia (Figure 2):^27^ “While having common cerebral lesions, the almost identical amino acid sequence of β protein of the cerebrovascular fibrils in both AD and adult DS is the first chemical evidence that DS is a pathologic model for AD.”^28^

Moreover, the homology between the Aβ protein found in cerebral vessels (cerebrovascular amyloidosis [CVA], now known as cerebral amyloid angiopathy [CAA]) and that found in neuritic plaques suggested a common origin: “The localization of the β protein antibodies to neuritic plaques in both AD and DS indicates that the source of amyloid in both plaques and vessels is the same.”^28^

**FIGURE 3:**
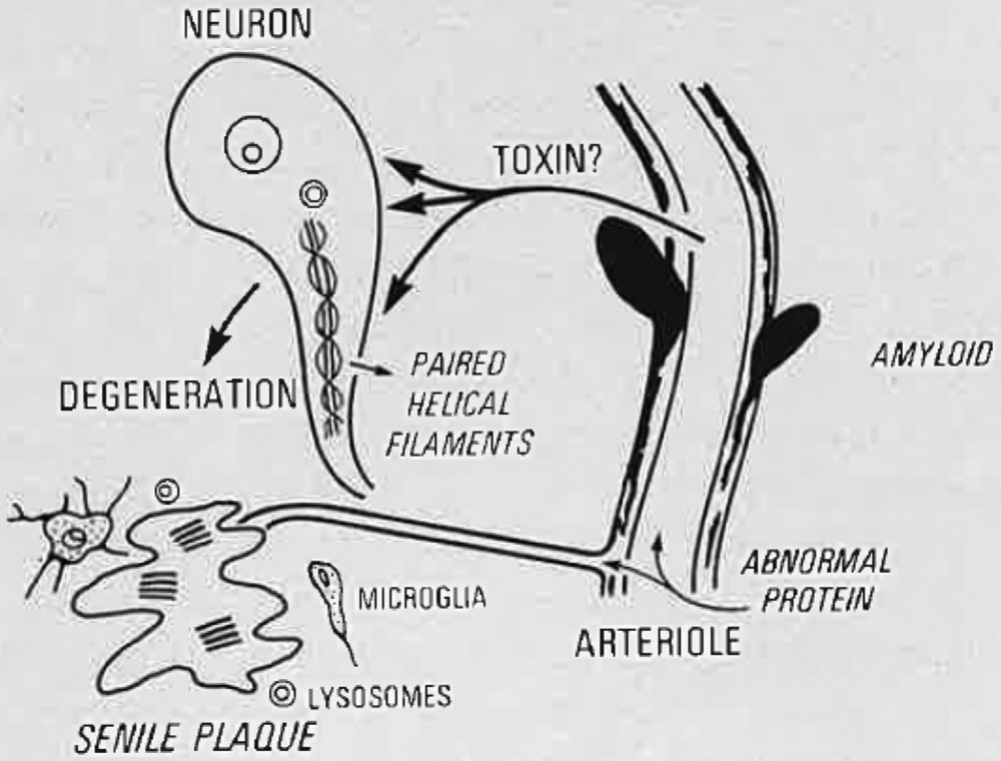
Image taken from Glenner et al, 1984,^28^ with permission from Elsevier.

However, the origin of these Aβ proteins and the relationship with the pathogenesis of neuritic plaque formation and AD remained unknown. Initially, Glenner et al. proposed that it could derive from an abnormal serum protein precursor, as was the case for other amyloidosis. This precursor would be specifically taken up by cerebrovascular endothelial cells to be proteolytically cleaved by their lysosomal complement to form amyloid fibrils that would damage capillary walls (CAA), causing the Aβ protein and other plasma proteins to enter the brain through breakage of the blood–brain barrier. This would lead to the formation of the amyloid of the neuritic plaque via lysosomal enzyme activity of microglia (Figure 3).^28^

In the quest to uncover the origin of this protein, its universal existence in individuals with DS likely prompted the consideration of a genetic cause: “Assuming the beta protein is a human gene product, it also suggests that the genetic defect in Alzheimer's disease is localized on chromosome 21.”^27^

An important breakthrough occurred in 1987 with the identification of the major protein subunit (Aβ) of the amyloid fibrils involved in AD both in non‐DS and DS patients by Kang et al.^29^ They described it as an insoluble, highly aggregating small polypeptide likely of neuronal origin and the cleavage product of a larger precursor protein. The sequence of this amyloid precursor protein (APP) was localized to the proximal portion of the long arm of chromosome 21 by four independent groups (Kang et al.,^29^ Goldgaber et al.,^30^ Robakis et al.,^31^ and Tanzi et al.^32^).

Whether the etiology of AD in the general population involved an increased dosage of the gene coding for the Aβ protein became a focal point of research, which greatly stimulated the genetic study of multiple families affected by the disease in the ensuing years. This led to the subsequent discovery of mutations in the *APP* gene^33^, ^34^ and in other related genes, such as apolipoprotein E,^35^, ^36^ presenilin 1,^37^, ^38^ and presenilin 2,^39^ the two latter related to the sequential cleavage of APP and forming, together with *APP* mutations and DSAD, the cornerstone of the amyloid cascade hypothesis.^40^

In DSAD, the relation between an increase in the dosage of the *APP* gene with AD and CAA was furthermore supported by rare autopsy findings in individuals with partial trisomy 21 in which *APP* was not overexpressed, showing an absence of AD neuropathology or CAA even at the very advanced ages (for the DS population) of 72 or 78 years.^41^, ^42^ These findings supported a necessary role for increased *APP* gene dose, one manifestation of which is an increase in the APP protein and its products, in driving the pathological features of AD in DS and motivated subsequent research to explore the early events leading to these changes. Pathological studies such as the work by Lemere et al. in 1996^43^ provided significant insights into the biochemistry and natural history of the disease. They demonstrated that diffuse plaques composed of Aβ42 begin to form in the brains of individuals with DS as early as 12 years of age. Later, other Aβ species, primarily Aβ40, were found to accumulate around the third decade of life, along with neuritic dystrophy and neurofibrillary tangles. The subsequent years focused on understanding the initial events in the formation of these lesions and the temporal changes leading to AD neuropathology in DS.^44^

Building on these foundational discoveries, DSAD research has continued to uncover key mechanisms underlying the pathogenesis of AD. For instance, alterations in the endocytic pathway are observed in cortical neurons of infants and even fetuses with DS, detectable before significant Aβ deposition, highlighting this pathway as one of the earliest intracellular manifestations of AD.^45^, ^46^ Additionally, research in DSAD has shed light on the timeline and potential contributions of various processes, including intracellular Aβ trafficking,^47^ lysosomal dysfunction,^45^ neurodegeneration of basal forebrain cholinergic neurons, nerve growth factor (NGF) dysregulation,^48^, ^49^ inflammation, and CAA in the development of AD.^50^, ^51^

Subsequent to these advances, numerous animal models for DS have been developed, predominantly in mice but not exclusively. While these models have yet to replicate the classical neuritic plaques characteristic of DSAD,^52^ they do exhibit tau pathology and effectively capture many other key features. Notably, research has demonstrated over the years the role of the full‐length APP protein, its C‐terminal fragments (CTFs), and A*β* species in contributing to endolysosomal (ELN) dysfunction and degenerative phenotypes^53^, ^54^ in both the Ts65Dn and Dp16 mouse models.^54^, ^55^ Promising developments include DS rats carrying a humanized *APP* gene (Yann Herault), which present significant potential for future research. Furthermore, DS‐derived induced pluripotent stem cell studies and organoids are emerging as innovative tools that may provide deeper insights into the complex interplay between DS and AD.

### 3.2 Clinical picture: the natural history of DSAD

From the first descriptions of a cognitive and functional decline among older individuals with DS by Jervis (1948) to the late 1970s, few clinico‐pathological cases of DSAD had yet been reported: “Although the neuropathological studies are in general agreement about the relationship between these two disorders, the proportion of individuals with Down's Syndrome who can be said to dement and have the features of Alzheimer's disease has not been as well established (…) In general, the possible clinical consequences of Alzheimer's change in the brains of those with Down's Syndrome has not been well documented.”^25^

With the growing biochemical and pathological evidence of DSAD as a genetic form of AD, an initial effort was made during the last decades of the twentieth century to delineate the clinical presentation, course, and diagnosis of the disease to understand its prevalence and natural history.[Fig alz70158-fig-0004]

A growing number of studies began focusing on the psychological and clinical signs associated with aging among the DS population. Most of them observed consistent findings of predominant behavioral changes with aging, later progressing to loss of self‐care skills and complete helplessness.^25^ However, comparing it to the pattern of initial cognitive decline followed by progressive dementia in AD in the general population, these descriptions in DS individuals could coincide with the later stage of the disease, preceded by undetected cognitive symptoms: “The lack of previous information on cognitive performance, combined with the great variability on cognitive testing of people with Down's Syndrome (Breg, 1977), makes it difficult to infer a previous performance level and therefore to recognize the signs of deterioration”^25^

A notable milestone was the initial description of the natural history of DSAD by Helen Evenhuis in 1990,^56^ in which she evaluated 17 individuals with DS in a prospective longitudinal study until the time of death, enabling a detailed description of the clinical evolution and later correlation with neuropathological findings. Hers and previous studies deepened the knowledge of the clinical picture of DSAD: “the natural history of dementia in patients with DS corresponds to that of DAT in patients without DS with respect to symptoms of cognitive and behavioral decline, but early stages are difficult to recognize.” This underscored the need for early and specific clinical evaluation: “it is feasible to achieve a clinical diagnosis of dementia in patients with DS by careful observations in daily life, with a comparison of former and actual levels of psychosocial functioning.”^56^ Notably, her work also commented on prevalent clinical symptoms among individuals with DSAD like gait and speech impairment as well as epilepsy and established a mean age of clinical onset of 51.3 to 52.6 years, which will align with future studies on the matter.^57^

Given the various degrees of baseline capacities and ID levels, one of the major difficulties was the application of neurological and cognitive tests among this population. Thus, an enormous effort was made during the subsequent years to develop accurate instruments for diagnosis, such as the adaption of the Cambridge Examination for Mental Disorders of the Elderly informant interview and the Cambridge Cognitive Examination neuropsychological battery,^58^, ^59^ to describe the rise of cognitive decline among individuals with DSAD.

**FIGURE 4 alz70158-fig-0004:**
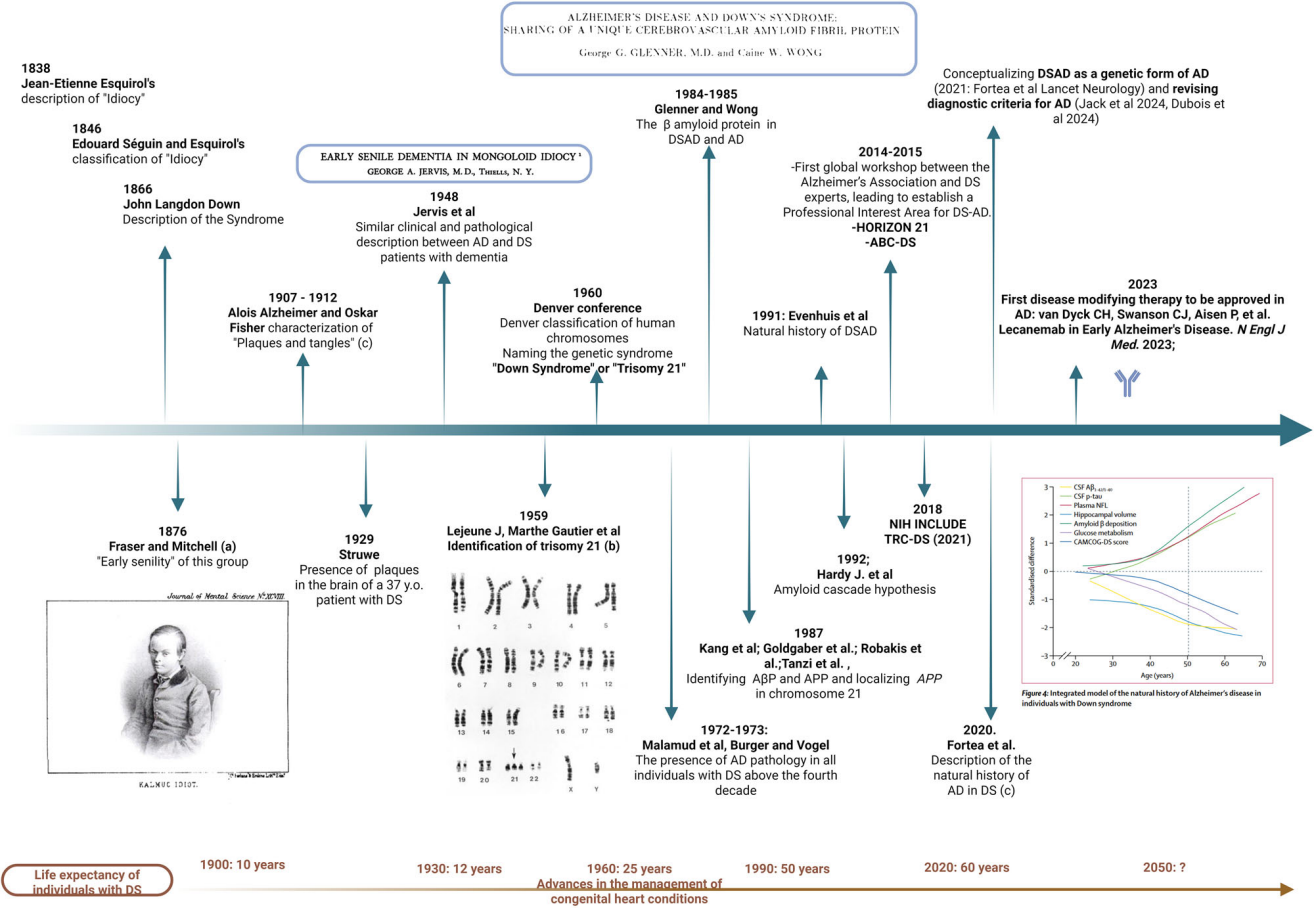
Historical overview of DSAD in the past 200 years. Created in BioRender. Maure blesa, L. (2025) https://BioRender.com/m92j537. All papers mentioned are references in the reference section. Images: (A) Drawing of a person with DS in the paper by Fraser and Mitchell. (B) Karyotype of an adult with DS. (C) Natural history figure from Fortea et al paper 2020. AD, Alzheimer's disease; APP, amyloid precursor protein; DS, Down syndrome; DSAD, Down syndrome–associated Alzheimer's disease.

These pivotal milestones of the 1990s laid the groundwork for the initial consideration of DSAD as a genetic form of the disease and catalyzed the subsequent research advancements that unfolded in the subsequent decades (Figure 4).

### 3.3 The impact of advocacy and deinstitutionalization in DS

When talking about how DS contributed to the characterization of AD, it is crucial to acknowledge that the study of adults with DS was only possible when individuals with DS began to experience longer life expectancy and were increasingly included in both the social and medical communities. This transformation was largely driven by parental advocacy, which played a fundamental role in challenging outdated perceptions and pushing for systemic change.^60^ For much of the twentieth century, most individuals with DS were transferred to institutions and many were deemed “uneducable,” effectively isolating them from both education and broader society. However, through the relentless efforts of parents and advocacy groups, institutions were improved, community living became a reality, and education for individuals with IDs, including DS, became more accessible and integrated. This not only contributed to individuals with DS receiving better medical care—leading to a significant increase in life expectancy from an average of 10 years in the early 1900s to > 60 years today—but also enabled them to receive a better education, fostering greater social participation and inclusion. As individuals with DS became more active members of society, opportunities for long‐term medical and cognitive research expanded, ultimately allowing scientists to uncover the knowledge we have today.

## 4 SHIFTING THE LENS: THE EVOLUTION OF APPROACHES TO MEDICAL RESEARCH IN DS

DSAD is now recognized as the largest group of people with genetically determined AD with near full penetrance.^61^, ^62^ There has been unprecedented increased attention and funding, leading to the creation of international consortia and enabling clinical trials to treat AD in this population. We briefly summarize some of the major milestones leading to this new research landscape over the past 25 years.

### 4.1 From pioneers to international consortia: overcoming barriers to inclusion

Unethical research involving individuals with IDs in the 1960s and 1970s revealed serious violations of ethical principles, leading to the implementation of stricter regulations to protect vulnerable populations. These measures were grounded in ethical frameworks such as the Nuremberg Code (1947) and the Declaration of Helsinki, which established informed consent as a cornerstone of research ethics. However, in seeking to prevent further exploitation, these regulations inadvertently excluded individuals with IDs from research, limiting their access to potential benefits. As a result, the tension between protection and inclusion became increasingly evident, reinforcing the need to address these barriers to ensure equitable participation in scientific progress.

In recent decades, and particularly over the past 10 years, there has been a notable move toward modern research ethics emphasizing the inclusion of these individuals in studies, ensuring equitable access to medical advancements alongside the general population.^63^, ^64^ This transformation is evident in the progression from the pioneering efforts of individual researchers in the 1980s and 1990s to expansive global collaborations that bring together researchers, clinicians, and advocacy groups.

The field of AD research changed starting in the first decade of the twenty‐first century with the enactment of NAPA (National Alzheimer's Project Act), which led to a remarkable increase in funding for AD to address the ambitious 2025 goal of achieving the first cure. Inspired initially by the harmonization of the Alzheimer's Disease Research Centers and National Alzheimer's Coordinating Center experiences, the NAPA identified several research gaps (https://aspe.hhs.gov/collaborations‐committees‐advisory‐groups/napa) and launched a request for application to address them inspired by research programs like the Alzheimer's Disease Neuroimaging Initiative, which transformed the approach to collaborative research, and later through initiatives like the Dominantly Inherited Alzheimer Network (DIAN) and the DIAN Trials Unit. This led to the Neurodegeneration in Aging Down Syndrome and Alzheimer's Disease in Down Syndrome initiatives that later converged into the Alzheimer Biomarker Consortium–Down Syndrome (ABC‐DS) consortium.^65^

Further increased funding from the National Institutes of Health (NIH) through the Investigation of Co‐occurring Conditions across the Lifespan to Understand Down Syndrome project, launched by the NIH in 2018, which brought additional targeted funding for DS research at large, and DSAD in particular, together with the work of the Alzheimer's Association, charities including Global Down Syndrome Foundation, LuMind Foundation, Jérôme Lejeune Foundation, and the Catalan Down Syndrome Foundation, among others, have converged to profoundly reshape the state of the field. These efforts have led to initiatives such as the Horizon 21 Consortium, established in Europe in 2014, the Down Syndrome Biobank Consortium, the ABC‐DS, and the Alzheimer's Clinical Trials Consortium–Down Syndrome (ACTC‐DS) (Table 1 ), that have collectively advanced research in this area by standardizing diagnostic criteria, creating harmonized protocols, and pooling longitudinal data and biomaterials from international sites.

**TABLE 1:** Observational studies of cognition and biomarkers in people with DS.

Observational studies			
	Sites	Objectives	Variables
Down Alzheimer Barcelona Neuroimaging Initiative (DABNI) https://santpaumemoryunit.com/alzheimer‐down‐unit/dabni‐down‐alzheimer‐barcelona‐neuroimaging‐initiative/	Population‐based cohort of Catalonia (Spain) centralized in a university tertiary hospital	To study the natural history of AD in DS through biomarkers; to recruit a trial‐ready cohort	Longitudinal clinical and cognitive data; biofluid biomarkers; genetics; neuroimaging biomarkers; sleep studies; EEG; brain banking
Down syndrome biomarker initiative (DSBI)^66^	One university, clinical and research centers in the United States	To characterize cognitive performance, neuroimaging, and plasma‐based AD biomarkers in a cohort of non‐demented adults with DS	Longitudinal clinical and cognitive data; biofluid biomarkers; genetics; neuroimaging biomarkers
The LonDowns Consortium https://www.kcl.ac.uk/research/london‐down‐syndrome‐consortium‐londowns	Four university, clinical, and research centers in the UK	To explore the cognitive, genetic, and cellular factors underlying individual differences in susceptibility to AD; to study individual differences in cognitive abilities and brain activity	Longitudinal clinical and cognitive data; biofluid biomarkers; genetics; neuroimaging biomarkers
Horizon 21 consortium https://horizon‐21.org/horizon‐21‐consortium/	10 university, clinical, and research centers in the UK	To identify factors that influence AD development in persons with DS; to develop clinical trials to prevent and/or slow the disease in this population	Longitudinal clinical and cognitive data; biofluid biomarkers; genetics; neuroimaging biomarkers; sleep studies
Alzheimer's Biomarkers Consortium–Down Syndrome (ABC‐DS) https://abc‐ds.org/ https://www.nia.nih.gov/research/abc‐ds	19 academic centers in the United States and UK	To identify biomarkers of cognitive impairment and dementia; to identify critical factors that link cerebral amyloid deposition to neurodegeneration and dementia; to understand the relationships between biomarkers; to provide rapid public access to all data	Longitudinal clinical and cognitive data; biofluids biomarkers; neuroimaging biomarkers; genetics
Alzheimer's Clinical Trials Consortium–Down Syndrome (ACTC‐DS) https://www.actc‐ds.org/	14 academic, clinical, and research sites in three countries (United States, UK, and Spain)	To analyze the relationships between cognitive measures and biomarkers of AD; to identify endpoints for AD clinical trials in DS that best reflect disease progression	Longitudinal cognitive and clinical assessment; biofluid biomarkers; genetics; neuroimaging biomarkers
LuMind IDSC Longitudinal Investigation for the Enhancement of Down Syndrome Research (LIFE‐DSR) https://dsctn.org/life‐dsr/	10 academic centers in the United States	To better understand the cognitive and behavioral changes along with the health issues found in adults with DS as they progress toward AD	Longitudinal health exam; blood biomarkers

Abbreviations: AD, Alzheimer's disease; DS, Down syndrome; EEG, electroencephalogram.

### 4.2 Bridging the gap in DSAD research

These combined efforts and initiatives have led to unprecedented advances in the field of DSAD. From more comprehensive clinical and neuropsychological assessments for AD, including adapted cognitive assessment,^67^ biomarker studies including blood and cerebrospinal fluid (CSF) biomarkers, magnetic resonance imaging, nuclear medicine scans,^68^, ^69^ and studies of neuropathology.^70^ Individuals with DS have a demonstrated willingness to participate in research if offered the opportunity.^71^, ^72^, ^73^ These efforts have led to significant progress in understanding the natural history of the disease,^74^ identifying many of the changes that precede the onset of clinical symptoms,^50^, ^74^ and establishing the utility of key biomarkers for diagnosing and prognosticating the disease.

Longitudinal studies from consortia and cohorts with extensive follow‐up have indeed further clarified the clinical presentation, including the estimated age of symptom onset 53.8 years (95% confidence interval: 53.1–54.5 years)^57^, ^75^ and factors that may play a role in it^76^, ^77^ as well as the clinical presentation of DSAD, which frequently resembles that of the typical presentation of sporadic AD with early episodic memory and executive deficits, in contrast to previous assumptions.^78^ Furthermore, the penetrance or lifetime risk of AD > 90% by the seventh decade of life, ultimately leading to the recognition of DSAD as a genetic form of AD and reflecting it in the updated criteria,^61^, ^78^, ^79^ akin to autosomal dominant AD (ADAD), with which it shares most characteristics and is studied similarly using concepts such as estimated year of onset.^57^ Overall, our understanding of DSAD has significantly expanded, and with it, the broader comprehension of AD as well.^46^

### 4.3 Clinical trials in DS: an opportunity we have yet to fully leverage

The future of care for this population must focus on preventing the disease, which is currently the leading cause of mortality and the main reason why life expectancy of those with DS has plateaued.^57^ However, until recently, there have been very few preventive and therapeutic clinical trials specifically designed for individuals with DS. No individual with DS has been included in the passive immunization trials against amyloid and current appropriate use recommendations of the approved disease‐modifying therapies advise against its use in DSAD due to the more frequent and severe CAA pathology associated with DSAD compared to sporadic AD,^80^, ^81^ a feature again shared in ADAD. This has prompted a debate in the field on how to navigate the conflicting concern for safety with the moral imperative to treat AD in this population.^63^, ^82^

Several clinical trials have been conducted in DSAD, demonstrating the feasibility of such studies (Table 2). However, their number remains limited, despite the significant opportunities and urgent need for therapeutics in this area.^83^ Among the most promising ongoing clinical trials targeting the amyloid cascade in DSAD are the ACTC‐DS–affiliated studies ABATE and HERO.

**TABLE 2:** Clinical trials of pharmacological interventions regarding DSAD in the last two decades.

	Study type	Setting	Eligibility criteria	Investigational agent	Primary endpoint	Reference
NCT01594346	36‐month randomized, double‐blind, placebo‐controlled phase 3 trial	Recruitment from 21 sites with research experience in adults with DS in five countries (Australia, Canada, Ireland, UK, and United States)	Adults with DS aged ≥ 50 years (*n* = 337)	Alpha‐tocopherol (vitamin E) versus placebo	Rate of change on the Brief Praxis Test	Sano et al. 2016^1^
NCT01791725 (DS 201)	4‐week randomized, double‐blind, placebo‐controlled, phase 2 trial	Three centers in the United States	Adults with DS without dementia (*n* = 26)	ELND005 (scyllo‐inositol;cyclohexane‐1,2,3,4,5,6‐hexol) versus placebo	Safety and tolerability	Rafii et al. 2017^2^
NCT02738450	12‐month placebo‐controlled, phase 1b multicenter study plus 12‐month follow‐up	Four centers in the United States	Adults with DS aged 25–45 years (*n* = 16)	ACI‐24 (liposomal vaccine against aggregated Aβ peptides) versus placebo	Safety and tolerability, antibody titer	Rafii et al. 2022^3^
NCT05462106 (recruiting)	Phase 1b/2, multicenter, double‐blind, randomized, placebo‐controlled	Nineteen centers in the United States, Spain, and UK	The phase 2 of this study is directed for adults with DS aged ≥ 35 and ≤ 50 at screening and PET scan at screening consistent with the presence of amyloid pathology and mild to moderate ID. This study is also open for prodromal AD in the general population (phase 1b: see inclusion criteria at ClinicalTrials.gov)	ACI‐24.060 (liposomal vaccine against aggregated Aβ peptides) at two different doses versus placebo	Safety, tolerability, immunogenicity, and pharmacodynamic effects of ACI‐24.060 in subjects with prodromal AD and in adults with DS (ABATE)	
NCT06673069 HERO (recruiting)	Phase 1b multi‐center, open‐label, SAD study in adult participants with DS with evidence of brain amyloid positivity	Ten sites in different countries	Adults with DS aged 35 to 55, cognitively stable, with an intelligence quotient (IQ) ≥ 45 and evidence of amyloid pathology on PET scan	ION269: investigational antisense oligonucleotide (ASO) designed to reduce the production of APP	Safety and tolerability of ION269 in adults with DS with evidence of brain amyloid positivity (HERO)	
NCT06206824 LEUCETTA (recruiting)	Randomized, double‐blind, placebo‐controlled phase 1 study to evaluate safety, tolerability, PK/PD of SAD, MAD, and food effect of leucettinib‐21 in healthy male subjects, and single dose in subjects with DSAD	One center in France	The study has four parts; part 4 includes individuals with DS and individuals with AD	Leucettinib‐21, investigational drug developed by Perha Pharmaceuticals, inspired by a natural compound from the marine sponge *Leucetta microraphis*. It is designed to inhibit the activity of the DYRK1A enzyme	Safety and tolerability of an oral administration of leucettinib‐21	

Abbreviations: Aβ, amyloid beta; AD, Alzheimer's disease; APP, amyloid precursor protein; DS, Down syndrome; DSAD, Down syndrome–associated Alzheimer's disease; ID, intellectual disability; MAD, multiple ascending dose; PET, positron emission tomography; PK/PD, pharmacokinetics/pharmacodynamics; SAD, single ascending dose.

The ABATE trial (AC Immune) is a phase 1b/2 study evaluating the anti‐amyloid vaccine ACI‐24.060 in both prodromal AD and DSAD. The HERO trial (Ionis) is a phase 1b study testing ION269, an antisense oligonucleotide targeting the *APP* gene. Additionally, the ACTC‐DS trial ALADDIN, a forthcoming phase 4 study (not yet registered), will assess the safety and tolerability of donanemab in individuals with DS.

Other clinical trials exploring alternative mechanisms in DSAD are also beginning to emerge, such as leucetta (see Table 2). These developments mark the beginning of a new era in the treatment of AD within this population.

## 5 CONCLUSION: A PATH FORWARD FOR DSAD

“The true measure of any society can be found in how it treats its most vulnerable members” (attributed to Gandhi, n.d.). The intrinsic human nature of care and inclusion, evident even in prehistoric and ancient societies that embraced and supported individuals with DS, has led to a much more active role of individuals with DS in our society, now sometimes living independent lives and with full participation in our society. This should include their active participation in responsible medical research. This ethos should inspire modern approaches to DSAD, emphasizing equity and scientific rigor.

There are many reasons for optimism about DSAD research. Recent advances in research, diagnostics, and therapeutic strategies have brought unprecedented progress. To unlock the full potential of DSAD research we need to continue and expand this coordinated effort across multiple fronts.^63^ Future directions should focus on enhancing awareness and involvement among the medical and general community; overcoming health inequities;^63^ and establishing robust research frameworks to further clarify the unique pathophysiology of DSAD, enhance biomarker discovery, and develop targeted interventions. Advocacy efforts must amplify the voices of individuals with DS and their families, ensuring their inclusion in policy and research decisions. Legislative measures are urgently needed to simplify and standardize the regulatory landscape for clinical trials in DS, reducing barriers and encouraging greater participation.

By fostering collaboration among researchers, policy makers, and advocacy groups we can move closer to developing effective treatments for DSAD. With a unified approach, the next decade holds the promise of transforming the prospect for individuals with DSAD and their families.

## CONFLICT OF INTEREST STATEMENT

The authors declare no conflicts of interest related to this study. Author disclosures are available in the supporting information.

## CONSENT STATEMENT

Consent was not necessary for this manuscript.

## Supporting information



Supporting Information

Supporting Information

## References

[1] Conde‐Valverde M , Quirós‐Sánchez A , Diez‐Valero J , et al. The child who lived: Down syndrome among Neanderthals?. Sci Adv. 2024;10(26):eadn9310. 38924400 10.1126/sciadv.adn9310 PMC11204207

[2] Rohrlach AB , Rivollat M , de‐Miguel‐Ibáñez P , et al. Cases of trisomy 21 and trisomy 18 among historic and prehistoric individuals discovered from ancient DNA. Nat Commun. 2024;15(1):1294. 38378781 10.1038/s41467-024-45438-1 PMC10879165

[3] Brown RI , Kyrkou MR , Watchman K , Hodapp RM . A brief history of down syndrome: key moments and reflections. In: Burack JA , Edgin JO , Abbeduto L , eds. The Oxford Handbook of Down Syndrome and Development. Oxford University Press; 2023. doi:10.1093/oxfordhb/9780190645441.013.1

[4] Starbuck JM . On the antiquity of trisomy 21: moving towards a quantitative diagnosis of Down syndrome in historic material culture. J Contemp Anthropol. 2011;2(1). https://hdl.handle.net/1805/4298

[5] Esquirol JED . In: Chez J‐B , ed. Des Maladies Mentales Considérées Sous les Rapports Médical, Hygiénique et Médico‐Légal. Baillière; 1838:712. PMC5093814 29918508

[6] Seguin É . Traitement Moral, Hygiène et Éducation Des Idiots et des Autres Enfants Arrièrés ou Rétardés Dans Leur Développement, Etc. Paris: J.B. Baillière; 1846:786.

[7] Tremblay JM . Mémoire et Rapport sur Victor de l'Aveyron. Chicoutimi, QC: UQAC; 2003. Available from: https://classiques.uqam.ca/classiques/itard_jean/victor_de_l_Aveyron/victor_preface_folliot.html

[8] Down JLH . Observations on an ethnic classification of idiots. J Ment Sci. 1867;13(61):121‐123. 7707939

[9] Wilkins RH , Brody IA . Down's syndrome. Arch Neurol. 1971;25(1):88‐90. 4259793 10.1001/archneur.1971.00490010098013

[10] Fraser J , Mitchell A . Kalmuc idiocy: report of a case with autopsy. J Ment Sci. 1876;22(98):169‐179.

[11] Tjio JH , Levan A . The chromosome number of man. Hereditas. 1956;42(1‐2):1‐6.

[12] Lejeune J , Gautier M , Turpin R . Study of somatic chromosomes from 9 mongoloid children. Comptes Rendus Hebd Seances Acad Sci. 1959;248(11):1721‐1722. 13639368

[13] Jacobs PA , Baikie AG , Court Brown WM , Strong JA . The somatic chromosomes in mongolism. Lancet Lond Engl. 1959;1(7075):710. 10.1016/s0140-6736(59)91892-6 13642857

[14] Book JA , Fraccaro M , Lindsten J . Cytogenetical observations in mongolism. Acta Paediatr. 1959;48:453‐468. 10.1111/j.1651-2227.1959.tb16409.x 13802656

[15] Allen G , Benda CE , Böök JA , et al. Mongolism. Am J Hum Genet. 1961;13(4):426. 17948460 PMC1932135

[16] Alzheimer A , Stelzmann RA , Schnitzlein HN , Murtagh FR . An english translation of Alzheimer's 1907 paper, ‘Uber eine eigenartige Erkankung der Hirnrinde’. Clin Anat N Y N. 1995;8(6):429‐431. 10.1002/ca.980080612 8713166

[17] Ryan NS , Rossor MN , Fox NC . Alzheimer's disease in the 100 years since Alzheimer's death. Brain. 2015;138(12):3816‐3821. 26541346 10.1093/brain/awv316

[18] Goedert M . Oskar Fischer and the study of dementia. Brain. 2009;132(4):1102‐1111. 18952676 10.1093/brain/awn256 PMC2668940

[19] Struwe F . Histopathologische Untersuchungen fiber Entstebung und Wesen der senilen Plaques. Z Gesamte Neurol Psychiatr. 1929;122:291–307.

[20] Jervis GA . Early senile dementia in mongoloid idiocy. Am J Psychiatry. 1948;105(2):102‐106. 18876430 10.1176/ajp.105.2.102

[21] Malamud N . Neuropathology of Organic Brain Syndromes Associated with Aging. In: Gaitz CM , ed. Aging and the Brain: The Proceedings of the Fifth Annual Symposium held at the Texas Research Institute of Mental Sciences in Houston, October 1971. Boston, MA: Springer US; 1995:63–87. doi:10.1007/978-1-4684-8503-5_6

[22] Burger PC , Vogel FS . The development of the pathologic changes of Alzheimer's disease and senile dementia in patients with Down's syndrome. Am J Pathol. 1973;73(2):457‐476. 4271339 PMC1904076

[23] Wisniewski KE , Wisniewski HM , Wen GY . Occurrence of neuropathological changes and dementia of Alzheimer's disease in Down's syndrome. Ann Neurol. 1985;17(3):278‐282. 3158266 10.1002/ana.410170310

[24] Heston LL . The genetics of Alzheimer's disease: associations with hematologic malignancy and Down's syndrome. Arch Gen Psychiatry. 1977;34(8):976. 142459 10.1001/archpsyc.1977.01770200114017

[25] Oliver C , Holland AJ . Down's syndrome and Alzheimer's disease: a review. Psychol Med. 1986;16(2):307‐322. 2941815 10.1017/s0033291700009120

[26] Glenner GG , Wong CW . Alzheimer's disease: initial report of the purification and characterization of a novel cerebrovascular amyloid protein. Biochem Biophys Res Commun. 1984;120(3):885‐890. 6375662 10.1016/s0006-291x(84)80190-4

[27] Glenner GG , Wong CW . Alzheimer's disease and Down's syndrome: sharing of a unique cerebrovascular amyloid fibril protein. Biochem Biophys Res Commun. 1984;122(3):1131‐1135. 6236805 10.1016/0006-291x(84)91209-9

[28] Glenner GG , Wong CW , Quaranta V , Eanes ED . The amyloid deposits in Alzheimer's disease: their nature and pathogenesis. Appl Pathol. 1984;2(6):357‐369. 6242724

[29] Kang J , Lemaire HG , Unterbeck A , et al. The precursor of Alzheimer's disease amyloid A4 protein resembles a cell‐surface receptor. Nature. 1987;325(6106):733‐736. 2881207 10.1038/325733a0

[30] Goldgaber D , Lerman MI , McBride WO , Saffiotti U , Gajdusek DC . Isolation, characterization, and chromosomal localization of human brain cDNA clones coding for the precursor of the amyloid of brain in Alzheimer's disease, Down's syndrome and aging. J Neural Transm Suppl. 1987;24:23‐28. 2960782

[31] Robakis NK , Wisniewski HM , Jenkins EC , et al. Chromosome 21q21 sublocalisation of gene encoding beta‐amyloid peptide in cerebral vessels and neuritic (senile) plaques of people with Alzheimer disease and Down syndrome. Lancet Lond Engl. 1987;1(8529):384‐385. 10.1016/s0140-6736(87)91754-5 2880184

[32] Tanzi RE , Gusella JF , Watkins PC , et al. Amyloid beta protein gene: cDNA, mRNA distribution, and genetic linkage near the Alzheimer locus. Science. 1987;235(4791):880‐884. 2949367 10.1126/science.2949367

[33] Goate A , Chartier‐Harlin MC , Mullan M , et al. Segregation of a missense mutation in the amyloid precursor protein gene with familial Alzheimer's disease. Nature. 1991;349(6311):704‐706. 1671712 10.1038/349704a0

[34] Murrell J , Farlow M , Ghetti B , Benson MD . A mutation in the amyloid precursor protein associated with hereditary Alzheimer's disease. Science. 1991;254(5028):97‐99. 1925564 10.1126/science.1925564

[35] Eh C , Am S , Wj S , et al. Gene dose of apolipoprotein E type 4 allele and the risk of Alzheimer's disease in late onset families. Science. 1993;261(5123):921‐923. https://pubmed.ncbi.nlm.nih.gov/8346443/ 8346443 10.1126/science.8346443

[36] Strittmatter WJ , Saunders AM , Schmechel D , et al. Apolipoprotein E: high‐avidity binding to beta‐amyloid and increased frequency of type 4 allele in late‐onset familial Alzheimer disease. Proc Natl Acad Sci U S A. 1993;90(5):1977‐1981. 8446617 10.1073/pnas.90.5.1977 PMC46003

[37] Sherrington R , Rogaev EI , Liang Y , et al. Cloning of a gene bearing missense mutations in early‐onset familial Alzheimer's disease. Nature. 1995;375(6534):754‐760. 7596406 10.1038/375754a0

[38] Rogaev EI , Sherrington R , Rogaeva EA , et al. Familial Alzheimer's disease in kindreds with missense mutations in a gene on chromosome 1 related to the Alzheimer's disease type 3 gene. Nature. 1995;376(6543):775‐778. 7651536 10.1038/376775a0

[39] Levy‐Lahad E , Wasco W , Poorkaj P , et al. Candidate gene for the chromosome 1 familial Alzheimer's disease locus. Science. 1995;269(5226):973‐977. 7638622 10.1126/science.7638622

[40] Hardy JA , Higgins GA . Alzheimer's disease: the amyloid cascade hypothesis. Science. 1992;256(5054):184‐185. 1566067 10.1126/science.1566067

[41] Doran E , Keator D , Head E , et al. Down syndrome, partial trisomy 21, and absence of Alzheimer's disease: the role of APP. J Alzheimers Dis. 2017;56(2):459‐470. 27983553 10.3233/JAD-160836 PMC5662115

[42] Prasher VP , Farrer MJ , Kessling AM , et al. Molecular mapping of Alzheimer‐type dementia in Down's syndrome. Ann Neurol. 1998;43(3):380‐383. 9506555 10.1002/ana.410430316

[43] Lemere CA , Blusztajn JK , Yamaguchi H , Wisniewski T , Saido TC , Selkoe DJ . Sequence of deposition of heterogeneous amyloid beta‐peptides and APO E in Down syndrome: implications for initial events in amyloid plaque formation. Neurobiol Dis. 1996;3(1):16‐32. 9173910 10.1006/nbdi.1996.0003

[44] Head E , Lott IT , Wilcock DM , Lemere CA . Aging in Down syndrome and the development of Alzheimer's disease neuropathology. Curr Alzheimer Res. 2016;13(1):18‐29. 26651341 10.2174/1567205012666151020114607 PMC4948181

[45] Nixon RA , Cataldo AM , Mathews PM . The endosomal‐lysosomal system of neurons in Alzheimer's disease pathogenesis: a review. Neurochem Res. 2000;25(9‐10):1161‐1172. 11059790 10.1023/a:1007675508413

[46] Maure‐Blesa L , Rodríguez‐Baz I , Carmona‐Iragui M , Fortea J . What can we learn about Alzheimer's disease from people with Down syndrome? Curr Top Behav Neurosci. 2025;69:197‐226. doi:10.1007/7854_2024_546 39509049

[47] Busciglio J , Pelsman A , Wong C , et al. Altered metabolism of the amyloid beta precursor protein is associated with mitochondrial dysfunction in Down's syndrome. Neuron. 2002;33(5):677‐688. 11879646 10.1016/s0896-6273(02)00604-9

[48] Pentz R , Iulita MF , Ducatenzeiler A , et al. Nerve growth factor (NGF) pathway biomarkers in Down syndrome prior to and after the onset of clinical Alzheimer's disease: a paired CSF and plasma study. Alzheimers Dement. 2021;17(4):605‐617. 33226181 10.1002/alz.12229 PMC8043977

[49] Bruno MA , Cuello AC . Activity‐dependent release of precursor nerve growth factor, conversion to mature nerve growth factor, and its degradation by a protease cascade. Proc Natl Acad Sci U S A. 2006;103(17):6735‐6740. 16618925 10.1073/pnas.0510645103 PMC1458950

[50] Lott IT , Head E . Dementia in Down syndrome: unique insights for Alzheimer disease research. Nat Rev Neurol. 2019;15(3):135‐147. 30733618 10.1038/s41582-018-0132-6 PMC8061428

[51] Carmona‐Iragui M , Videla L , Lleó A , Fortea J . Down syndrome, Alzheimer disease, and cerebral amyloid angiopathy: the complex triangle of brain amyloidosis. Dev Neurobiol. 2019;79(7):716‐737. 31278851 10.1002/dneu.22709

[52] Rueda N , Flórez J , Dierssen M , Martínez‐Cué C . Translational validity and implications of pharmacotherapies in preclinical models of Down syndrome. Prog Brain Res. 2020;251:245‐268. 32057309 10.1016/bs.pbr.2019.10.001

[53] Salehi A , Delcroix JD , Belichenko PV , et al. Increased APP expression in a mouse model of Down's syndrome disrupts NGF transport and causes cholinergic neuron degeneration. Neuron. 2006;51(1):29‐42. 16815330 10.1016/j.neuron.2006.05.022

[54] Sawa M , Overk C , Becker A , et al. Impact of increased APP gene dose in Down syndrome and the Dp16 mouse model. Alzheimers Dement. 2022;18(6):1203‐1234. 34757693 10.1002/alz.12463 PMC9085977

[55] Xu W , Weissmiller AM , White JA , et al. Amyloid precursor protein‐mediated endocytic pathway disruption induces axonal dysfunction and neurodegeneration. J Clin Invest. 2016;126(5):1815‐1833. 27064279 10.1172/JCI82409 PMC4855914

[56] Evenhuis HM . The natural history of dementia in Down's syndrome. Arch Neurol. 1990;47(3):263‐267. 2138013 10.1001/archneur.1990.00530030029011

[57] Iulita MF , Garzón Chavez D , Klitgaard Christensen M , et al. Association of Alzheimer disease with life expectancy in people with Down syndrome. JAMA Netw Open. 2022;5(5):e2212910. 35604690 10.1001/jamanetworkopen.2022.12910 PMC9127560

[58] Hon J , Huppert FA , Holland AJ , Watson P . Neuropsychological assessment of older adults with Down's syndrome: an epidemiological study using the Cambridge Cognitive Examination (CAMCOG). Br J Clin Psychol. 1999;38(2):155‐165. 10389597 10.1348/014466599162719

[59] Holland AJ , Hon J , Huppert FA , Stevens F . Incidence and course of dementia in people with Down's syndrome: findings from a population‐based study. J Intellect Disabil Res JIDR. 2000;44(Pt 2):138‐146. 10898377 10.1046/j.1365-2788.2000.00263.x

[60] George S . Parental Advocacy and the Changing Attitudes Towards Down syndrome in Post‐war Britain . 2020. [cited 2025 Feb 26].

[61] Jack CR , Andrews JS , Beach TG , et al. Revised criteria for diagnosis and staging of Alzheimer's disease: Alzheimer's Association Workgroup. Alzheimers Dement. 2024;20(8):5143‐5169. 38934362 10.1002/alz.13859 PMC11350039

[62] Dubois B , Villain N , Schneider L , et al. Alzheimer Disease as a clinical‐biological construct—an International Working Group Recommendation. JAMA Neurol. 2024;81(12):1304‐1311. https://jamanetwork.com/journals/jamaneurology/fullarticle/2825806 39483064 10.1001/jamaneurol.2024.3770 PMC12010406

[63] Fortea J , McGlinchey E , Espinosa JM , Rafii MS . Addressing challenges in health care and research for people with Down syndrome. Lancet Lond Engl. 2024;403(10439):1830‐1833. 10.1016/S0140-6736(24)00478-1 38521088

[64] Hartley D , Blumenthal T , Carrillo M , et al. Down syndrome and Alzheimer's disease: common pathways, common goals. Alzheimers Dement. 2015;11(6):700‐709. 25510383 10.1016/j.jalz.2014.10.007 PMC4817997

[65] Handen BL , Lott IT , Christian BT , et al. The Alzheimer's biomarker consortium‐Down Syndrome: rationale and methodology. Alzheimers Dement. 2020;12(1):e12065. 10.1002/dad2.12065 PMC7396809 32775597

[66] Rafii MS , Wishnek H , Brewer JB , et al. The down syndrome biomarker initiative (DSBI) pilot: proof of concept for deep phenotyping of Alzheimer's disease biomarkers in down syndrome. Front Behav Neurosci. 2015;9:239. doi:10.3389/fnbeh.2015.00239 26441570 PMC4568340

[67] Videla L , Benejam B , Pegueroles J , et al. Longitudinal clinical and cognitive changes along the Alzheimer disease continuum in Down syndrome. JAMA Netw Open. 2022;5(8):e2225573. 35930282 10.1001/jamanetworkopen.2022.25573 PMC9356319

[68] Landt J , D'Abrera JC , Holland AJ , et al. Using positron emission tomography and Carbon 11‐labeled Pittsburgh compound B to image brain fibrillar β‐amyloid in adults with down syndrome: safety, acceptability, and feasibility. Arch Neurol. 2011;68(7):890‐896. 21403005 10.1001/archneurol.2011.36

[69] Hendrix JA , Airey DC , Britton A , et al. Cross‐sectional exploration of plasma biomarkers of Alzheimer's disease in Down syndrome: early data from the longitudinal investigation for enhancing down syndrome research (LIFE‐DSR) study. J Clin Med. 2021;10(9):1907. 33924960 10.3390/jcm10091907 PMC8124643

[70] Martini AC , Gross TJ , Head E , Mapstone M . Beyond amyloid: immune, cerebrovascular, and metabolic contributions to Alzheimer disease in people with Down syndrome. Neuron. 2022;110(13):2063‐2079. 35472307 10.1016/j.neuron.2022.04.001 PMC9262826

[71] Carmona‐Iragui M , Santos T , Videla S , et al. Feasibility of lumbar puncture in the study of cerebrospinal fluid biomarkers for Alzheimer's Disease in subjects with down syndrome. J Alzheimer's Dis. 2017;55(4), 1489‐1496. doi:10.3233/JAD-160827 27858714

[72] Fortea J , Carmona‐Iragui M , Benejam B , et al. Plasma and CSF biomarkers for the diagnosis of Alzheimer's disease in adults with Down syndrome: a cross‐sectional study. Lancet Neurol. 2018;17(10):860‐869. 30172624 10.1016/S1474-4422(18)30285-0

[73] Neale N , Padilla C , Fonseca LM , Holland T , Zaman S . Neuroimaging and other modalities to assess Alzheimer's disease in Down syndrome. NeuroImage Clin. 2018;17:263‐271. 29159043 10.1016/j.nicl.2017.10.022 PMC5683343

[74] Fortea J , Vilaplana E , Carmona‐Iragui M , et al. Clinical and biomarker changes of Alzheimer's disease in adults with Down syndrome: a cross‐sectional study. Lancet Lond Engl. 2020;395(10242):1988‐1997. 10.1016/S0140-6736(20)30689-9 PMC7322523 32593336

[75] Larsen FK , Baksh RA , McGlinchey E , et al. Age of Alzheimer's disease diagnosis in people with Down syndrome and associated factors: results from the Horizon 21 European Down syndrome consortium. Alzheimers Dement. 2024;20(5):3270‐3280. 38506627 10.1002/alz.13779 PMC11095427

[76] Snyder HM , Bain LJ , Brickman AM , et al. Further understanding the connection between Alzheimer's disease and Down syndrome. Alzheimers Dement. 2020;16(7):1065‐1077. 32544310 10.1002/alz.12112 PMC8865308

[77] Iulita MF , Bejanin A , Vilaplana E , et al. Association of biological sex with clinical outcomes and biomarkers of Alzheimer's disease in adults with Down syndrome. Brain Commun. 2023;5(2):fcad074. 37056479 10.1093/braincomms/fcad074 PMC10088472

[78] Dubois B , Feldman HH , Jacova C , et al. Advancing research diagnostic criteria for Alzheimer's disease: the IWG‐2 criteria. Lancet Neurol. 2014;13(6):614‐629. 24849862 10.1016/S1474-4422(14)70090-0

[79] Fortea J , Zaman SH , Hartley S , Rafii MS , Head E , Carmona‐Iragui M . Alzheimer's disease associated with Down syndrome: a genetic form of dementia. Lancet Neurol. 2021;20(11):930‐942. 34687637 10.1016/S1474-4422(21)00245-3 PMC9387748

[80] Head E , Phelan MJ , Doran E , et al. Cerebrovascular pathology in Down syndrome and Alzheimer disease. Acta Neuropathol Commun. 2017;5(1):93. 29195510 10.1186/s40478-017-0499-4 PMC5709935

[81] Liu L , Saba A , Pascual JR , et al. Lecanemab and vascular‐amyloid deposition in brains of people with Down syndrome. JAMA Neurol. 2024;81(10):1066‐1072. 39158850 10.1001/jamaneurol.2024.2579 PMC11334015

[82] Rafii MS , Fortea J . Down syndrome in a new era for Alzheimer disease. JAMA. 2023;330(22):2157‐2158. 37991807 10.1001/jama.2023.22924 PMC11324235

[83] Strydom A , Coppus A , Blesa R , et al. Alzheimer's disease in Down syndrome: an overlooked population for prevention trials. Alzheimers Dement N Y N. 2018;4:703‐713. 10.1016/j.trci.2018.10.006 PMC6296162 30581976

