# Supplementary material for: The history of Down syndrome–associated Alzheimer's disease; past, present, and future
Source: Alzheimers Dement. 2025 Jun 5;21(6):e70158. doi: 10.1002/alz.70158 (PMC12138279; doi:10.1002/alz.70158)

Understanding Down Syndrome and Alzheimer’s Disease: A Journey Through History

Introduction

Over the years, individuals with Down syndrome (DS) have been central to important medical discoveries. One of the most significant findings is their connection to Alzheimer’s disease (AD). As individuals with DS age, AD becomes a major challenge in their lives. At the same time, their condition provides a crucial model for studying and understanding the disease.

A Look at the Past

Although people with DS have existed since the beginning of human history, it was only officially identified and recognized as a distinct form of intellectual disability (ID) in the late 19th century. In the past, many individuals with DS were placed in institutions and were not given the opportunity to go to school or receive proper medical care. However, in the mid 20th century, families and advocates of individuals with DS led a movement to change this. They fought for better education, medical attention, and community inclusion. This movement allowed people with DS to live longer and healthier lives, and ultimately gave scientists the chance to study how they age—including how and why they develop AD.

How Down Syndrome Helped Scientists Understand Alzheimer’s Disease

Early on, doctors noticed that people with DS seemed to experience memory problems and dementia at young ages. By studying their brains, scientists found that they had the same changes seen in people with AD. Eventually, researchers discovered that this was due to an extra copy of chromosome 21, which carries a gene involved in the production of a protein called amyloid-beta. This protein builds up in the brain leading to memory loss and other symptoms of AD.

Thanks to research in DS, scientists were able to uncover many of the key mechanisms behind AD, helping not just the DS community but also millions of people worldwide who are at risk for the disease

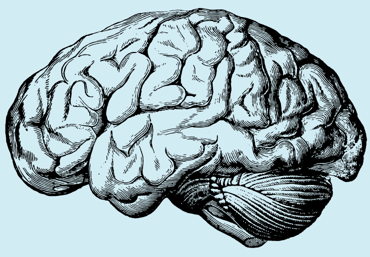

Overcoming Barriers: Proving That Medical Assessments Work in Individuals with DS

For many years, there was a misconception that individuals with DS and other intellectual disabilities could not reliably participate in clinical assessments or medical research. However, research has proven otherwise. Scientists have developed specialized tests that accurately measure cognitive changes in people with DS, allowing for better diagnosis and monitoring of AD.
Additionally, individuals with DS have shown to be willing and able to participate in medical evaluations, including complex procedures like brain imaging and biomarker testing. With the right support and adapted approaches, people with DS can actively contribute to research and benefit from medical advancements. This has been a major breakthrough in ensuring they are included in clinical trials for potential AD treatments.

The Future: What's Next for DS and AD Research?

Today, people with DS are included in major research efforts on AD. Scientists are working on new treatments and ways to slow or even prevent the disease. Thanks to international collaborations, clinical trials are now being designed specifically for individuals with DS, ensuring they have access to the latest medical advances.

However, challenges remain. More work is needed to ensure individuals with DS are included in research and have access to the best possible care. Advocacy continues to play a crucial role in pushing for these changes.

Conclusion: The Power of Inclusion

The story of DS and AD research is one of progress and resilience. What we know today about AD would not have been possible without individuals with DS and the families who fought for their rights. By continuing to prioritize inclusion, advocacy, and research, we can improve the lives of people with DS while also advancing our understanding of one of the most challenging diseases of aging.

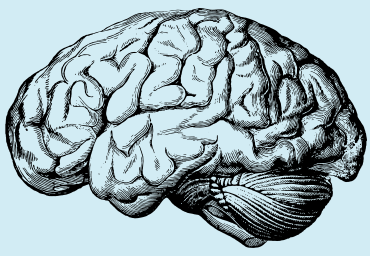

Supplement: Supplementary file 1 — Supporting Information [file ALZ-21-e70158-s002.pdf]
